# AIM2 Inflammasome's First Decade of Discovery: Focus on Oral Diseases

**DOI:** 10.3389/fimmu.2020.01487

**Published:** 2020-08-13

**Authors:** Lufei Wang, Lu Sun, Kevin M. Byrd, Ching-Chang Ko, Zhenxing Zhao, Jie Fang

**Affiliations:** ^1^State Key Laboratory of Oral Diseases, National Clinical Research Center for Oral Diseases, Department of Orthodontics, West China Hospital of Stomatology, Sichuan University, Chengdu, China; ^2^Division of Oral and Craniofacial Health Sciences, University of North Carolina Adams School of Dentistry, Chapel Hill, NC, United States; ^3^Department of Oral Medicine, Infection and Immunity, Harvard School of Dental Medicine, Boston, MA, United States; ^4^Division of Orthodontics, The Ohio State University College of Dentistry, Columbus, OH, United States

**Keywords:** innate immunity, inflammasome, periodontal disease, pulpitis, head and neck cancers

## Abstract

A common feature of many acute and chronic oral diseases is microbial-induced inflammation. Innate immune responses are the first line of defense against pathogenic microorganisms and are initiated by pattern recognition receptors (PRRs) that specifically recognize pathogen-associated molecular patterns and danger-associated molecular patterns. The activation of certain PRRs can lead to the assembly of macromolecular oligomers termed *inflammasomes*, which are responsible for pro-inflammatory cytokine maturation and secretion and thus activate host inflammatory responses. About 10 years ago, the absent in melanoma 2 (AIM2) was independently discovered by four research groups, and among the “canonical” inflammasomes [including AIM2, NLR family pyrin domain (NLRP)1, NLRP3, NLR family apoptosis inhibitory protein (NAIP)/NLR family, caspase activation and recruitment domain (CARD) containing (NLRC)4, and pyrin], AIM2 so far is the only one that simultaneously acts as a cytosolic DNA sensor due to its DNA-binding ability. Undoubtedly, such a double-faceted role gives AIM2 greater mission and more potential in the mediation of innate immune responses. Therefore, AIM2 has garnered much attention from the broad scientific community during its first 10 years of discovery (2009–2019). How the AIM2 inflammasome is related to oral diseases has aroused debate over the past few years and is under active investigation. AIM2 inflammasome may potentially be a key link between oral diseases and innate immunity. In this review, we highlight the current knowledge of the AIM2 inflammasome and its critical role in the pathogenesis of various oral diseases, which might offer future possibilities for disease prevention and targeted therapy utilizing this continued understanding.

## Introduction

A common feature of many oral diseases is microbial-induced inflammation. For example, periodontal disease, which is a disease characterized by a microbial dysbiosis-triggered inflammatory response in a susceptible host, has been reported to afflict ~50% of all US adults (1). Underlying the oral inflammatory diseases are innate immune responses, which are the first line of defense against pathogenic microorganisms and are initiated by pattern recognition receptors (PRRs) that specifically recognize pathogen-associated molecular patterns (PAMPs) and danger-associated molecular patterns (DAMPs) (2). PRRs are an important component of the innate immune system and are essential for the induction of immune reactions against microbial infection. The activation of certain PRRs can lead to the assembly of large multiprotein complexes termed inflammasomes, which are responsible for the release of pro-inflammatory cytokines that contribute to the development of inflammatory diseases (3).

The understanding of how oral tissues activate inflammatory pathways in response to environmental challenges is important for precise therapies. In current oral and craniofacial disease research, inflammasomes are being investigated in oral inflammatory diseases, oral autoimmune diseases, and head and neck squamous cell carcinoma. It has been 10 years since the discovery of the absent in melanoma 2 (AIM2) inflammasome (2009–2019). Thus, in this review, we highlight the current knowledge on the AIM2 inflammasome and its critical role in the pathogenesis of various oral diseases, which might offer directions for the next 10 years.

## Cytosolic DNA Sensors

**Figure 1:**
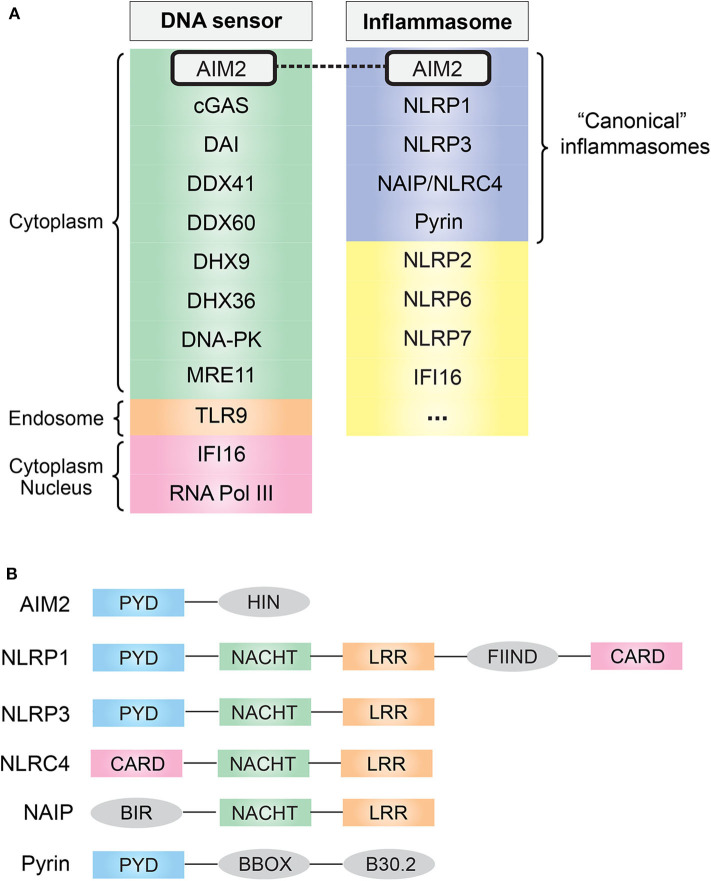
**(A)** Common cytosolic DNA sensors and inflammasomes. AIM2 simultaneously acts as a cytosolic DNA sensor and an inflammasome. **(B)** Domain and structural difference. Common domain PYD and CARD are essential for the assembly of inflammasome: PYD domain can recruit ASC via PYD–PYD interaction, and CARD domain can recruit caspase-1 via CARD–CARD interaction. AIM2, absent in melanoma 2; cGAS, cyclic-GMP-AMP synthase; DAI, DNA-dependent activator of interferon regulatory factor; DDX, DEAD-box helicase; DHX, DEAH-box helicase; DNA-PK, DNA-dependent protein kinase; MRE11, MRE11 homolog, double-strand break repair nuclease; TLR9, Toll-like receptor 9; IFI16, IFN-γ inducible protein 16; RNA Pol III, RNA polymerase III; NLR, nucleotide-binding domain and leucine-rich repeat containing; NAIP, NLR family apoptosis inhibitory protein; PYD, pyrin domain; NACHT, nucleotide-binding oligomerization domain; LRR, leucine-rich repeat; CARD, caspase activation and recruitment domain; FIIND, function-to-find domain; BIR, baculovirus inhibitor of apoptosis repeat; BBOX, two B-box zinc finger.

In eukaryotes, DNA is confined to the nucleus and mitochondria during homeostasis; however, the exposure of DNA in cytosol usually suggests either an active infection (pathogen DNA) or DNA damage. The discovery of Toll-like receptor 9 (TLR9) first demonstrated the existence of innate immune receptors that directly respond to DNA (4). DNA is now known to be recognized by many innate immune receptors that reside in the cytoplasm or endolysosomal compartment (Figure 1A). They generally converge on adaptor protein stimulator of interferon (IFN) genes (STING), leading to the transcription of type I IFN via the STING–TANK binding kinase 1 (TBK1)–IFN regulatory factor (IRF) 3/7 axis (5).

DNA recognition by innate immune receptors triggers the activation of multiple pathways that collectively act as a “double-edged sword” to the host: protective but also detrimental (6). The best-characterized pathway activated by DNA recognition is the expression of type I IFN via the STING–TBK1–IRF3/7 axis, which mainly contributes to host antiviral responses (7). Other well-characterized DNA-stimulated pathways include nuclear factor kappa light chain enhancer of activated B cells (NF-κB) pathway that drives the expression of many pro-inflammatory genes and the inflammasome pathways that lead to the maturation of interleukin (IL)-1β/IL-18 (8).

## AIM2 Inflammasome

As one of the downstream effects of DNA sensing, the assembly of a multi-protein complex termed inflammasome, which generally consists of specific PRRs, apoptosis-associated speck-like protein containing a CARD (ASC) and caspase-1, was found in 2002 (9). Inflammasomes provide a molecular platform for the activation of inflammatory caspases in response to danger signals, which eventually causes the secretion of pro-inflammatory cytokine IL-1β/IL-18 (3, 10). To date, several well-characterized “canonical” inflammasome complexes have been identified according to the specific PRR involved: AIM2, NLR family pyrin domain (NLRP)1, NLRP3, neuronal apoptosis inhibitory protein (NAIP)/NLR family, caspase activation and recruitment domain (CARD) containing (NLRC)4, and pyrin [(11); Figure 1A]. In addition, some other sensors have also been proposed to form inflammasomes, such as human IFN gamma-inducible protein 16 (IFI16), NLRP2 and NLRP7, and murine NLRP6 and NLRP9b (12), and it is likely that more inflammasome complexes are coming out soon.

Among the “canonical” inflammasomes, what makes AIM2 distinct from others is its DNA recognition ability resulting from a unique HIN domain (Figure 1B). This was first suspected from a 2008 study that found that transfection of viral/bacterial DNA can lead to inflammasome activation even in the absence of NLRP3, but both ASC and caspase-1 are required for this process, indicating the existence of a DNA-sensing inflammasome distinct from the known NLR family (13). One year later, in 2009, four research groups independently identified this DNA-sensing inflammasome protein, AIM2, by using different methods (14–17). Undoubtedly, the role of the DNA sensor plus inflammasome determines the fate of AIM2: a crucial sentinel in immune defense.

## AIM2 Inflammasome: Initialization, Assembly, and Downstream Effect

AIM2 consists of one N-terminal PYD domain and one C-terminal HIN domain (Figure 1B), which provides a structural basis for its function as a DNA-sensing inflammasome. IFI16 and other PYD and HIN domain-containing proteins are now identified as AIM2-like receptors (ALRs) family (18). Biochemical studies have demonstrated that AIM2 can directly bind DNA, and there are three major features of the AIM2–DNA interaction (19): (1) the interaction is HIN domain-dependent, as HIN domain has two oligonucleotide/oligosaccharide binding folds that have high affinity to DNA; (2) it is independent of DNA sequence, GC content, or origin but the DNA length must be at least 80 base pairs; (3) a preference for double-stranded DNA (dsDNA).

The PYD domain has a strong tendency to self-aggregate and can interact with other PYD-containing proteins via PYD–PYD interaction. cryo-EM structural analysis of the AIM2^PYD^ filament has suggested assembly plasticity in both AIM2^PYD^ and ASC^PYD^, which may explain the versatility in PYD–PYD interactions (20). Classically, it has been suggested that AIM2^HIN^ sequesters AIM2^PYD^ in resting state, whereas the binding of dsDNA will disrupt this auto-inhibition and liberate the PYD domain (21). This can be advantageous as the PYD–PYD interaction contributes to both the oligomerization of AIM2 and the subsequent recruitment of adaptor protein ASC. Soon after, ASC further recruits procaspase-1 via CARD–CARD domain interaction, leading to the final assembly of inflammasome complex (22, 23). Intriguingly, this auto-inhibition model has been challenged by a recent work that suggested that the role of AIM2^PYD^ is to oligomerize and drive filament formation, rather than to maintain auto-inhibition (24). Thus, the structural basis for the AIM2 inflammasome initialization requires further elucidation.

Once assembled, the AIM2 inflammasome complex further enables the proteolytic activity of caspase-1 on IL-1β, IL-18, and gasdermin D (GSDMD), leading to the maturation of these proteins. Of these, inflammatory cytokines IL-1β/IL-18 are capable of initiating the inflammatory cascade in a variety of tissues upon binding to its receptor, suggesting their important role in the development of inflammatory diseases (25, 26). GSDMD is typically involved in a pro-inflammatory type of cell death termed pyroptosis (27). GSDMD disrupts the plasma membrane upon the proteolytic activation by caspase-1, leading to lytic cell death and the release of DAMPs (28, 29).

## AIM2 Recognizes Host and Pathogen DNA

Host DNA (nuclear or mitochondrial) is released into the cytosol following damage of the membrane integrity resulting from pathological stimulations, which acts as a trigger in AIM2-dependent innate immunity (6, 30). For example, during cellular stress or carcinogenesis, the integrity of nuclear envelope is transiently disrupted, leading to the cytosolic presence of host DNA that is directly recognized by AIM2 (31). Mitochondrial DNA released during mitochondrial stress resulting from cholesterol overload has also been demonstrated to activate the AIM2 inflammasome (32). Additionally, AIM2 is also capable of sensing other types of host DNA, including DNA damage within the nucleus and self-DNA secreted by exosomes [reviewed in Lugrin and Martinon (22)].

**Figure 2:**
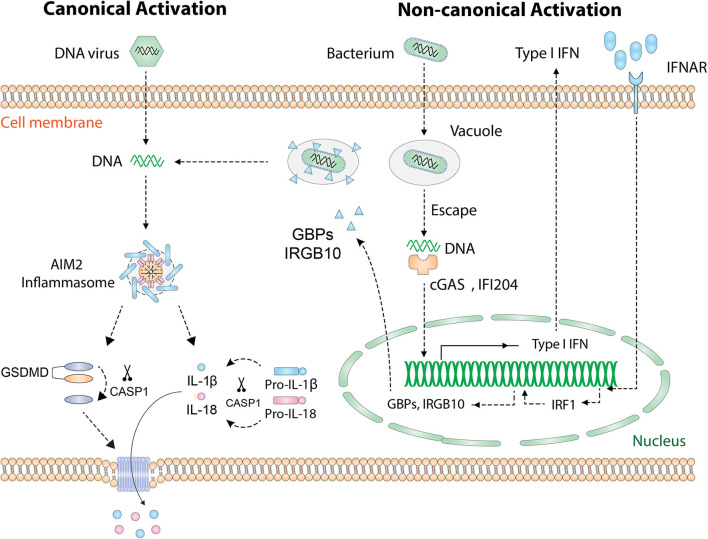
Activation of the AIM2 inflammasome. Cytosolic DNA [DNA virus, poly(dA:dT)] is directly recognized by AIM2 and leads to the “canonical” activation of AIM2 inflammasome. Cytosolic bacteria activate AIM2 inflammasome via a type I IFN-dependent “non-canonical” pathway. Intracellular bacteria escape the vacuole and release small amounts of DNA, which is detected by cGAS and IFI204. Then type I IFN is synthesized and drives the expression of IRF1 in an autocrine manner. Upon the expression of IRF1, GBP2/GBP5 and IRGB10 are produced and cause the rupture of bacterial/vacuolar membrane, exposing a mass of DNA for AIM2 to recognize. Finally, assembly of the AIM2 inflammasome causes proteolytic activation of IL-1β/IL-18 and GSDMD. GSDMD forms pores on the cell membrane that allow the secretion of IL-1β/IL-18. AIM2, absent in melanoma 2; GSDMD, gasdermin D; IFN, interferon; IFNAR, IFN α and β receptor; cGAS, cyclic-GMP-AMP synthase; IFI204, IFN activated gene 204; IRF1, IFN regulatory factor 1; GBP, guanylate-binding protein; IRGB10, immunity-related GTPase family member b10.

During microbial infection of a host cell, PAMP and microbial DNA are released into the cytoplasm and are detected by specific PRRs. In addition to recognizing host DNA, AIM2 recognizes DNA from intracellular pathogens including viruses and bacteria. Currently, there are two known activation pathways for AIM2 inflammasome (Figure 2): the “canonical” and “non-canonical” pathways (23). In cases of DNA virus infection or transfection of synthetic DNA analog poly(dA:dT), exposed dsDNA in the cytoplasm is directly recognized by AIM2 and hence initiates inflammasome assembly. This process is defined as the “canonical” activation, which is rapid and does not require the activity of type I IFN (33). Many DNA viruses, such as mouse cytomegalovirus, vaccinia virus, and human papillomavirus (HPV), activate AIM2 inflammasome in this way (34). Interestingly, not all DNA viruses can be recognized by AIM2, such as herpes simplex virus 1 (HSV-1), possibly because DNA–AIM2 interaction is inhibited during viral infection (35).

The “non-canonical” activation which requires type I IFN activity occurs during most bacterial infections (11). To date, several bacteria species, including *Listeria monocytogenes, Streptococcus pneumoniae, Porphyromonas gingivalis* (*P. gingivalis*), and *Staphylococcus aureus*, have been found to activate AIM2 inflammasome [thoroughly reviewed in Man et al. (23)]. Many groups are pursuing questions of how bacterial DNA is eventually recognized by AIM2. A proposed mechanism is that intracellular bacteria escape the vacuole and expose small amounts of DNA, leading to the activation of cyclic-GMP-AMP synthase (cGAS) and IFI204 that cooperatively drives the production of type I IFN (36). Interestingly, it is unknown why the released bacterial DNA cannot be recognized by AIM2 at this initial stage. Once activated, type I IFN is secreted outside the cell, binds to the type I IFN receptor (IFNR) in an autocrine fashion, and drives the expression of downstream guanylate-binding proteins (GBPs) and immunity-related GTPase family member b10 (IRGB10) via type I IFN/IRF1 axis. At this stage, abundant amounts of bacterial DNA are released due to GBP2, GBP5, and IRGB10-induced bacteriolysis and are eventually recognized by AIM2 (37–39).

Regardless of the “canonical” or “non-canonical” activation, the events downstream of AIM2 DNA sensing are the same (Figure 2): the assembly of inflammasome complex, the activation of caspase-1, and the maturation of IL-1β/IL-18 and GSDMD (22, 23, 40). Therefore, AIM2 is essential for the regulation of innate immune responses and plays a central role in the pathogenesis of inflammatory diseases, autoimmune diseases, and cancers.

## AIM2 Inflammasome and Oral Diseases

### Periodontitis

Periodontitis is a common chronic inflammatory disease that has a very high prevalence (30–50%) in adults (1). The onset and progression of periodontitis is closely associated with gram-negative anaerobic bacteria, such as *P. gingivalis, Aggregatibacter actinomycetemcomitans* (*A. actinomycetemcomitans*), and *Tannerella forsythia* (*T. forsythia*) (41, 42). Bacteria-triggered inflammasome activity plays an important role in the pathogenesis of periodontal diseases (43, 44). Recent research has found early evidence for the AIM2 inflammasome in a variety of periodontitis diagnoses. A genome-wide association study (GWAS) found that AIM2 and IFI16 single-nucleotide polymorphisms (SNPs) are associated with increased clinical parameters of periodontal disease, increased levels of gingival crevicular fluid IL-1β, and higher bacterial loads (45). Integrative analysis of GWAS and expression quantitative trait loci data further identified AIM2 as a susceptibility gene for periodontitis (46). The expression of AIM2 in the gingival tissues of chronic periodontitis patients has been confirmed (47, 48). Later, the expression of AIM2 has been confirmed in broader types of periodontitis gingival tissues, including chronic periodontitis, aggressive periodontitis, and gingivitis (49, 50).

While *P. gingivalis* can induce IL-1β secretion, the underlying mechanism is not clear: is it AIM2-dependent and what periodontal pathogens can activate AIM2 inflammasome? These questions are being pursued step by step: multi-species biofilm → individual bacterial species → virulence factors. Bostanci et al. (51) found that six-species supragingival biofilm increased the expression of AIM2, caspase-1, and IL-1β genes in gingival fibroblasts, whereas 10-species subgingival biofilm enhanced these gene expression at low concentrations but inhibited at high concentrations. Interestingly, Belibasakis et al. (52) found that neither complete 10-species subgingival biofilm nor the nine-species variant (excluding *P. gingivalis*) significantly affected the expression of AIM2 gene. A possible reason is that high concentrations of biofilm supernatants elicited equivalent cell responses to viable biofilms.

Later, Park et al. (48) found that an individual bacterial species—*P. gingivalis*—induces IL-1β secretion and pyroptosis in differentiated THP-1 monocytic cells. They further demonstrated that this phenomenon depends on both NLRP3 and AIM2 inflammasome activation and also requires the priming signal via TLR2/TLR4. However, a recent contradiction is that *P. gingivalis* exclusively activates NLRP3 rather than AIM2 inflammasome in bone marrow-derived macrophages (BMDMs): during *P. gingivalis* infection, NLRP3 deletion efficiently blocked IL-1β production, whereas AIM2 deletion did not suppress IL-1β production (53). In addition to *P. gingivalis, A. actinomycetemcomitans* has also been reported to induce IL-1β production in differentiated THP-1 cells via activating AIM2 inflammasome (54). A robust upregulation of AIM2 but not NLRP3 was detected during *A. actinomycetemcomitans* infection, suggesting that AIM2 dominantly affects the defense against *A. actinomycetemcomitans* infection that is also confirmed by AIM2 siRNA knockdown assay.

*Porphyromonas gingivalis* outer membrane vesicles (OMVs) have been proposed to induce inflammasome activation (55). Cecil et al. (56) found that in THP-1 cells, the OMVs of *P. gingivalis, T. denticola*, and *T. forsythia* can trigger the activation of NLRP3 and AIM2 inflammasomes and induce pro-inflammatory cytokine production through the NF-κB pathway. *P. gingivalis* OMVs even triggered inflammasome activation *in vivo*, as proved by IL-1β secretion and ASC speck formation (56). However, this is challenged by a recent report, which suggested that *P. gingivalis* OMVs are unlikely to contribute to the activation of inflammasomes (53). *P. gingivalis* mutant strain KDP361, which has a reduced amount of OMVs, did not affect IL-1β secretion in differentiated THP-1 cells compared to *P. gingivalis* wild-type strain. They also proposed that inflammasome activation upon *P. gingivalis* infection is independent of gingipains but is associated with the heat-labile components released from the bacteria (53). Collectively, the mechanism by which periodontal pathogens induce AIM2 inflammasome activity appears to be very complicated and awaits further elucidation.

### Endodontic Infections

The nature of endodontic infections is polymicrobial infections mostly comprised of gram-negative anaerobic bacteria (57). Pulpal and periapical inflammatory responses triggered by bacteria are responsible for a series of endodontic outcomes: from a simple reversible pulpitis to pulp necrosis and the eventual periapical lesion (58). During endodontic infections, inflammasomes function as a versatile machine for detecting pathogens and modulating inflammatory reactions in the root canal microenvironment (59).

The expression of AIM2 in rat or human pulpitis tissue has been reported separately but has a similar distribution pattern (60, 61): AIM2 was only expressed in the odontoblasts of healthy pulp tissue, whereas AIM2 was robustly expressed in the inflammatory cells and fibroblasts of pulpitis tissue. In the population of apical periodontitis, the expressions of AIM2 and NLRP3 were mainly detected in the inflammatory cells (neutrophils, macrophages, monocytes, and plasma cells) of periapical tissues (62).

Regarding the mechanism, it has been reported that cytoplasmic DNA activates AIM2 inflammasome and hence induces IL-1β release in human dental pulp cells (HDPCs) in a time- and dose-dependent manner (61). dsDNA plus IFN-γ stimulation enhanced AIM2/ASC/caspase-1 expression and IL-1β secretion, comparing to dsDNA only, which is a supportive evidence for the involvement of type I IFN-dependent AIM2 activation pathway in dental pulpitis (61). The central role of AIM2 in the modulation of dental pulp immune responses is further confirmed by overexpression/knockdown assay: AIM2 overexpression significantly increased caspase-1 activity and IL-1β secretion in HDPCs, whereas AIM2 knockdown decreased caspase-1 activity and IL-1β secretion, which collectively indicates that the AIM2 inflammasome pathway is required for caspase-1-dependent IL-1β secretion in HDPCs (63).

### Head and Neck Squamous Cell Carcinoma/Oral Squamous Cell Carcinoma

Head and neck squamous cell carcinoma (HNSCC) is the most common malignancy of the lip, oral cavity, and oropharynx. Because of the high recurrence rate and frequent distant metastases, the 5-year overall survival rate of oral squamous cell carcinoma (OSCC) patients is only around 50%, and several risk factors have been identified so far, such as smoking, alcohol consumption, and chronic inflammation (64). A high-density SNP array study on OSCC samples revealed high expression of AIM2 and IFI16 with frequent amplification at chromosome 1q23 (65). Similarly, a whole-genome sequencing study in an OSCC population with chewing-tobacco habit found significant upregulation of AIM2 in oral cancer tissues comparing to normal oral tissues (66). In HPV-positive or -negative HNSCC, IFI16 and AIM2 have been proposed as potential biomarkers for prognosis (67).

The underlying question that remains is as to what the underlying mechanism of AIM2 inflammasome OSCC initiation actually is. Although AIM2 was thought to be a tumor suppressor, overexpression of AIM2 and IFI16 synergistically promotes OSCC cell growth and prevents apoptosis via activating NF-κB pathway, which suggests the oncogenic potential of AIM2 and IFI16 in OSCC (65). Intriguingly, this co-expression of AIM2 and IFI16 promotes tumor cell growth only in the absence of p53, implying a possible cross-talk between p53 and NF-κB signaling pathway. AIM2 also contributes to OSCC progression, as overexpression of AIM2 in OSCC cell lines enhanced *in vitro* cell migration, invasion capacity, and epithelial–mesenchymal transition (68). When AIM2-overexpressing OSCC cells were implanted into the tongues of immunodeficient mice, enhanced tumor growth and lymphatic invasion were observed, resulting in a decreased survival rate (68). Collectively, AIM2 is implicated in both the development and progression of OSCC and may be a novel therapeutic target for OSCC.

### Other Oral Diseases

Early evidence has also been shown in other oral diseases, even though not much. The ductal salivary epithelia of primary Sjögren's syndrome patients display persistent AIM2 activation due to cytoplasmic accumulations of damaged DNA (69). A certain etiologic agent of dental caries, *Streptococcus mutans*, is capable of activating AIM2 inflammasome in THP-1 cells (70). HSV-1, which primarily causes oral herpes, inhibits AIM2 activation via tegument protein VP22 to enable immune evasion and lifelong latency (35).

## Aiming for the Next 10 Years and Beyond

As discussed above, current evidences regarding AIM2 inflammasome in oral diseases mainly focused on periodontal diseases, dental pulpitis, and OSCC. Aiming for the next 10 years and beyond, several open questions would be valuable to bear in mind. First, while previous studies detected the activation of AIM2 inflammasome in the pathogenesis of periodontitis and pulpitis, the molecular mechanism by which oral pathogens activate AIM2 has not yet been fully elucidated. Type I IFN-dependent “non-canonical” activation pathway, which is so far the best working model by which bacteria activate AIM2, may be a valuable reference for future studies. Second, it will be necessary to dissect the inflammasome-downstream effects (caspase-1 activation and IL-1β release) are actually due to which inflammasome. In particular, open questions that remain are what inflammatory reactions in oral diseases are AIM2-dependent, NLRP3-dependent, or both. Third, given the close relationship between inflammasome-mediated pyroptosis and infectious diseases or cancer, exploring AIM2/caspase-1/GSDMD axis in oral diseases will be of great importance to the current knowledge. In short, although further work is needed, delineating the pathological role of AIM2 inflammasome will undoubtedly advance the development of oral disease therapy.

## Author Contributions

LW and LS contributed to conception and design, data acquisition and interpretation, and drafted the manuscript. KB, C-CK, ZZ, and JF contributed to conception and design, and drafted and critically revised the manuscript. All authors gave final approval and agree to be accountable for all aspects of the work. All authors contributed to the article and approved the submitted version.

## Conflict of Interest

The authors declare that the research was conducted in the absence of any commercial or financial relationships that could be construed as a potential conflict of interest.

## References

[1] Eke PI Dye BA Wei L Slade GD Thornton-Evans GO Borgnakke WS. Update on prevalence of periodontitis in adults in the United States: NHANES 2009 to 2012. J Periodontol. (2015) 86:611–22. 10.1902/jop.2015.140520 25688694 PMC4460825

[2] Cao X. Self-regulation and cross-regulation of pattern-recognition receptor signalling in health and disease. Nat Rev Immunol. (2016) 16:35–50. 10.1038/nri.2015.8 26711677

[3] Rathinam VA Fitzgerald KA. Inflammasome complexes: emerging mechanisms and effector functions. Cell. (2016) 165:792–800. 10.1016/j.cell.2016.03.046 27153493 PMC5503689

[4] Hemmi H Takeuchi O Kawai T Kaisho T Sato S Sanjo H. A Toll-like receptor recognizes bacterial DNA. Nature. (2000) 408:740–5. 10.1038/35047123 11130078

[5] Chen Q Sun L Chen ZJ. Regulation and function of the cGAS-STING pathway of cytosolic DNA sensing. Nat Immunol. (2016) 17:1142–9. 10.1038/ni.3558 27648547

[6] Gallucci S Maffei ME. DNA Sensing across the Tree of Life. Trends Immunol. (2017) 38:719–32. 10.1016/j.it.2017.07.012 28886908

[7] Paludan SR Bowie AG. Immune sensing of DNA. Immunity. (2013) 38:870–80. 10.1016/j.immuni.2013.05.004 23706668 PMC3683625

[8] Abe T Marutani Y Shoji I. Cytosolic DNA-sensing immune response and viral infection. Microbiol Immunol. (2019) 63:51–64. 10.1111/1348-0421.12669 30677166 PMC7168513

[9] Martinon F Burns K Tschopp J. The inflammasome: a molecular platform triggering activation of inflammatory caspases and processing of proIL-beta. Mol Cell. (2002) 10:417–26. 10.1016/s1097-2765(02)00599-3 12191486

[10] Kesavardhana S Kanneganti TD. Mechanisms governing inflammasome activation, assembly and pyroptosis induction. Int Immunol. (2017) 29:201–10. 10.1093/intimm/dxx018 28531279 PMC5890894

[11] Hayward JA Mathur A Ngo C Man SM. Cytosolic recognition of microbes and pathogens: inflammasomes in action. Microbiol Mol Biol Rev. (2018) 82:e00015–18. 10.1128/MMBR.00015-18 30209070 PMC6298609

[12] Place DE Kanneganti TD. Recent advances in inflammasome biology. Curr Opin Immunol. (2018) 50:32–8. 10.1016/j.coi.2017.10.011 29128729 PMC5857399

[13] Muruve DA Petrilli V Zaiss AK White LR Clark SA Ross PJ. The inflammasome recognizes cytosolic microbial and host DNA and triggers an innate immune response. Nature. (2008) 452:103–7. 10.1038/nature06664 18288107

[14] Roberts TL Idris A Dunn JA Kelly GM Burnton CM Hodgson S. HIN-200 proteins regulate caspase activation in response to foreign cytoplasmic DNA. Science. (2009) 323:1057–60. 10.1126/science.1169841 19131592

[15] Fernandes-Alnemri T Yu JW Datta P Wu J Alnemri ES. AIM2 activates the inflammasome and cell death in response to cytoplasmic DNA. Nature. (2009) 458:509–13. 10.1038/nature07710 19158676 PMC2862225

[16] Hornung V Ablasser A Charrel-Dennis M Bauernfeind F Horvath G Caffrey DR. AIM2 recognizes cytosolic dsDNA and forms a caspase-1-activating inflammasome with ASC. Nature. (2009) 458:514–8. 10.1038/nature07725 19158675 PMC2726264

[17] Burckstummer T Baumann C Bluml S Dixit E Durnberger G Jahn H. An orthogonal proteomic-genomic screen identifies AIM2 as a cytoplasmic DNA sensor for the inflammasome. Nat Immunol. (2009) 10:266–72. 10.1038/ni.1702 19158679

[18] Caneparo V Landolfo S Gariglio M De Andrea M. The absent in melanoma 2-like receptor IFN-inducible protein 16 as an inflammasome regulator in systemic lupus erythematosus: the dark side of sensing microbes. Front Immunol. (2018) 9:1180. 10.3389/fimmu.2018.01180 29892303 PMC5985366

[19] Wang B Yin Q. AIM2 inflammasome activation and regulation: a structural perspective. J Struct Biol. (2017) 200:279–82. 10.1016/j.jsb.2017.08.001 28813641 PMC5733693

[20] Lu A Li Y Yin Q Ruan J Yu X Egelman E. Plasticity in PYD assembly revealed by cryo-EM structure of the PYD filament of AIM2. Cell Discov. (2015) 1:15013. 10.1038/celldisc.2015.13 26583071 PMC4646227

[21] Jin T Perry A Jiang J Smith P Curry JA Unterholzner L. Structures of the HIN domain:DNA complexes reveal ligand binding and activation mechanisms of the AIM2 inflammasome and IFI16 receptor. Immunity. (2012) 36:561–71. 10.1016/j.immuni.2012.02.014 22483801 PMC3334467

[22] Lugrin J Martinon F. The AIM2 inflammasome: sensor of pathogens and cellular perturbations. Immunol Rev. (2018) 281:99–114. 10.1111/imr.12618 29247998

[23] Man SM Karki R Kanneganti TD. AIM2 inflammasome in infection, cancer, and autoimmunity: Role in DNA sensing, inflammation, and innate immunity. Eur J Immunol. (2016) 46:269–80. 10.1002/eji.201545839 26626159 PMC4758349

[24] Morrone SR Matyszewski M Yu X Delannoy M Egelman EH Sohn J. Assembly-driven activation of the AIM2 foreign-dsDNA sensor provides a polymerization template for downstream ASC. Nat Commun. (2015) 6:7827. 10.1038/ncomms8827 26197926 PMC4525163

[25] Mantovani A Dinarello CA Molgora M Garlanda C. Interleukin-1 and related cytokines in the regulation of inflammation and immunity. Immunity. (2019) 50:778–95. 10.1016/j.immuni.2019.03.012 30995499 PMC7174020

[26] Kaneko N Kurata M Yamamoto T Morikawa S Masumoto J. The role of interleukin-1 in general pathology. Inflamm Regen. (2019) 39:12. 10.1186/s41232-019-0101-5 31182982 PMC6551897

[27] Kovacs SB Miao EA. Gasdermins: effectors of pyroptosis. Trends Cell Biol. (2017) 27:673–84. 10.1016/j.tcb.2017.05.005 28619472 PMC5565696

[28] Shi J Zhao Y Wang K Shi X Wang Y Huang H. Cleavage of GSDMD by inflammatory caspases determines pyroptotic cell death. Nature. (2015) 526:660–5. 10.1038/nature15514 26375003

[29] Ding J Wang K Liu W She Y Sun Q Shi J. Pore-forming activity and structural autoinhibition of the gasdermin family. Nature. (2016) 535:111–6. 10.1038/nature18590 27281216

[30] Kausar S Yang L Abbas MN Hu X Zhao Y Zhu Y. Mitochondrial DNA: a key regulator of anti-microbial innate immunity. Genes. (2020) 11:86. 10.3390/genes11010086 31940818 PMC7017290

[31] Di Micco A Frera G Lugrin J Jamilloux Y Hsu ET Tardivel A. AIM2 inflammasome is activated by pharmacological disruption of nuclear envelope integrity. Proc Natl Acad Sci USA. (2016) 113:E4671–80. 10.1073/pnas.1602419113 27462105 PMC4987819

[32] Dang EV McDonald JG Russell DW Cyster JG. Oxysterol restraint of cholesterol synthesis prevents AIM2 inflammasome activation. Cell. (2017) 171:1057–71.e11. 10.1016/j.cell.2017.09.029 29033131 PMC5693620

[33] Ma Z Ni G Damania B. Innate sensing of DNA virus genomes. Annu Rev Virol. (2018) 5:341–62. 10.1146/annurev-virology-092917-043244 30265633 PMC6443256

[34] Shrivastava G Leon-Juarez M Garcia-Cordero J Meza-Sanchez DE Cedillo-Barron L. Inflammasomes and its importance in viral infections. Immunol Res. (2016) 64:1101–17. 10.1007/s12026-016-8873-z 27699580

[35] Maruzuru Y Ichinohe T Sato R Miyake K Okano T Suzuki T. Herpes simplex virus 1 VP22 inhibits AIM2-dependent inflammasome activation to enable efficient viral replication. Cell Host Microbe. (2018) 23:254–65.e7. 10.1016/j.chom.2017.12.014 29447697

[36] Storek KM Gertsvolf NA Ohlson MB Monack DM. cGAS and Ifi204 cooperate to produce type I IFNs in response to Francisella infection. J Immunol. (2015) 194:3236–45. 10.4049/jimmunol.1402764 25710914 PMC4367159

[37] Man SM Karki R Sasai M Place DE Kesavardhana S Temirov J. IRGB10 liberates bacterial ligands for sensing by the AIM2 and caspase-11-NLRP3 inflammasomes. Cell. (2016) 167:382–96.e17. 10.1016/j.cell.2016.09.012 27693356 PMC5074697

[38] Man SM Karki R Malireddi RK Neale G Vogel P Yamamoto M. The transcription factor IRF1 and guanylate-binding proteins target activation of the AIM2 inflammasome by Francisella infection. Nat Immunol. (2015) 16:467–75. 10.1038/ni.3118 25774715 PMC4406811

[39] Meunier E Wallet P Dreier RF Costanzo S Anton L Ruhl S. Guanylate-binding proteins promote activation of the AIM2 inflammasome during infection with Francisella novicida. Nat Immunol. (2015) 16:476–84. 10.1038/ni.3119 25774716 PMC4568307

[40] Zhu W Zu X Liu S Zhang H. The absent in melanoma 2 (AIM2) inflammasome in microbial infection. Clin Chim Acta. (2019) 495:100–8. 10.1016/j.cca.2019.04.052 30959045

[41] Ebersole JL Dawson D III Emecen-Huja P Nagarajan R Howard K Grady ME. The periodontal war: microbes and immunity. Periodontol 2000. (2017) 75:52–115. 10.1111/prd.12222 28758303

[42] Kinane DF Stathopoulou PG Papapanou PN Periodontal diseases. Nat Rev Dis Primers. (2017) 3:17038 10.1038/nrdp.2017.38 28805207

[43] Aral K Milward MR Kapila Y Berdeli A Cooper PR. Inflammasomes and their regulation in periodontal disease: a review. J Periodontal Res. (2020) 10.1111/jre.12733. [Epub ahead of print]. 31960443

[44] Marchesan JT Girnary MS Moss K Monaghan ET Egnatz GJ Jiao Y. Role of inflammasomes in the pathogenesis of periodontal disease and therapeutics. Periodontol 2000. (2020) 82:93–114. 10.1111/prd.12269 31850638 PMC6927484

[45] Marchesan JT Jiao Y Moss K Divaris K Seaman W Webster-Cyriaque J. Common polymorphisms in IFI16 and AIM2 genes are associated with periodontal disease. J Periodontol. (2017) 88:663–72. 10.1902/jop.2017.160553 28387608 PMC5695043

[46] Li W Zheng Q Meng H Chen D. Integration of genome-wide association study and expression quantitative trait loci data identifies AIM2 as a risk gene of periodontitis. J Clin Periodontol. (2020) 47:583–93. 10.1111/jcpe.13268 32031269

[47] Sahingur SE Xia XJ Voth SC Yeudall WA Gunsolley JC. Increased nucleic Acid receptor expression in chronic periodontitis. J Periodontol. (2013) 84:e48–57. 10.1902/jop.2013.120739 23646855

[48] Park E Na HS Song YR Shin SY Kim YM Chung J. Activation of NLRP3 and AIM2 inflammasomes by *Porphyromonas gingivalis* infection. Infect Immun. (2014) 82:112–23. 10.1128/iai.00862-13 24126516 PMC3911849

[49] Xue F Shu R Xie Y. The expression of NLRP3, NLRP1 and AIM2 in the gingival tissue of periodontitis patients: RT-PCR study and immunohistochemistry. Arch Oral Biol. (2015) 60:948–58. 10.1016/j.archoralbio.2015.03.005 25841070

[50] Aral K Berdeli E Cooper PR Milward MR Kapila Y Karadede Unal B. Differential expression of inflammasome regulatory transcripts in periodontal disease. J Periodontol. (2020) 91:606–16. 10.1002/JPER.19-0222 31557327

[51] Bostanci N Meier A Guggenheim B Belibasakis GN. Regulation of NLRP3 and AIM2 inflammasome gene expression levels in gingival fibroblasts by oral biofilms. Cell Immunol. (2011) 270:88–93. 10.1016/j.cellimm.2011.04.002 21550598

[52] Belibasakis GN Guggenheim B Bostanci N. Down-regulation of NLRP3 inflammasome in gingival fibroblasts by subgingival biofilms: involvement of *Porphyromonas gingivalis*. Innate Immun. (2013) 19:3–9. 10.1177/1753425912444767 22522430

[53] Okano T Ashida H Suzuki S Shoji M Nakayama K Suzuki T. *Porphyromonas gingivalis* triggers NLRP3-mediated inflammasome activation in macrophages in a bacterial gingipains-independent manner. Eur J Immunol. (2018) 48:1965–74. 10.1002/eji.201847658 30280383

[54] Kim S Park MH Song YR Na HS Chung J. Aggregatibacter actinomycetemcomitans-induced AIM2 inflammasome activation is suppressed by xylitol in differentiated THP-1 macrophages. J Periodontol. (2016) 87:e116–26. 10.1902/jop.2016.150477 26876350

[55] Fleetwood AJ Lee MKS Singleton W Achuthan A Lee MC O'Brien-Simpson NM. Metabolic remodeling, inflammasome activation, and pyroptosis in macrophages stimulated by *Porphyromonas gingivalis* and its outer membrane vesicles. Front Cell Infect Microbiol. (2017) 7:351. 10.3389/fcimb.2017.00351 28824884 PMC5543041

[56] Cecil JD O'Brien-Simpson NM Lenzo JC Holden JA Singleton W Perez-Gonzalez A. Outer membrane vesicles prime and activate macrophage inflammasomes and cytokine secretion *in vitro* and *in vivo*. Front Immunol. (2017) 8:1017. 10.3389/fimmu.2017.01017 28890719 PMC5574916

[57] Fouad AF. Microbial factors and antimicrobial strategies in dental pulp regeneration. J Endod. (2017) 43:S46–50. 10.1016/j.joen.2017.06.010 28778502

[58] Persoon IF Ozok AR. Definitions and epidemiology of endodontic infections. Curr Oral Health Rep. (2017) 4:278–85. 10.1007/s40496-017-0161-z 29201596 PMC5688219

[59] Jang JH Shin HW Lee JM Lee HW Kim EC Park SH. An overview of pathogen recognition receptors for innate immunity in dental pulp. Mediators Inflamm. (2015) 2015:794143. 10.1155/2015/794143 26576076 PMC4630409

[60] Wang Y Zhai S Wang H Jia Q Jiang W Zhang X. Absent in melanoma 2 (AIM2) in rat dental pulp mediates the inflammatory response during pulpitis. J Endod. (2013) 39:1390–4. 10.1016/j.joen.2013.07.003 24139260

[61] Huang S Song Z Jiang L Chen L Wang R Qin W. Absent in melanoma 2 (AIM2) expressed in human dental pulp mediates IL-1beta secretion in response to cytoplasmic DNA. Inflammation. (2015) 38:566–75. 10.1007/s10753-014-9963-5 24986444

[62] Ran S Liu B Gu S Sun Z Liang J. Analysis of the expression of NLRP3 and AIM2 in periapical lesions with apical periodontitis and microbial analysis outside the apical segment of teeth. Arch Oral Biol. (2017) 78:39–47. 10.1016/j.archoralbio.2017.02.006 28193569

[63] Huang S Song Z Huang Q Jiang L Chen L Wang R. AIM2 inflammasome is critical for dsDNA-induced IL-1beta secretion in human dental pulp cells. Inflammation. (2018) 41:409–17. 10.1007/s10753-017-0697-z 29178062

[64] Shield KD Ferlay J Jemal A Sankaranarayanan R Chaturvedi AK Bray F. The global incidence of lip, oral cavity, and pharyngeal cancers by subsite in 2012. CA Cancer J Clin. (2017) 67:51–64. 10.3322/caac.21384 28076666

[65] Kondo Y Nagai K Nakahata S Saito Y Ichikawa T Suekane A. Overexpression of the DNA sensor proteins, absent in melanoma 2 and interferon-inducible 16, contributes to tumorigenesis of oral squamous cell carcinoma with p53 inactivation. Cancer Sci. (2012) 103:782–90. 10.1111/j.1349-7006.2012.02211.x 22320325 PMC7659186

[66] Chakrabarti S Multani S Dabholkar J Saranath D. Whole genome expression profiling in chewing-tobacco-associated oral cancers: a pilot study. Med Oncol. (2015) 32:60. 10.1007/s12032-015-0483-4 25663065

[67] Riva G Pecorari G Biolatti M Pautasso S Lo Cigno I Garzaro M PYHIN genes as potential biomarkers for prognosis of human papillomavirus-positive or -negative head and neck squamous cell carcinomas. Mol Biol Rep. (2019) 46:3333–47. 10.1007/s11033-019-04795-7 30980272

[68] Nakamura Y Nakahata S Kondo Y Izumi A Yamamoto K Ichikawa T. Overexpression of absent in melanoma 2 in oral squamous cell carcinoma contributes to tumor progression. Biochem Biophys Res Commun. (2019) 509:82–8. 10.1016/j.bbrc.2018.12.066 30587341

[69] Vakrakou AG Svolaki IP Evangelou K Gorgoulis VG Manoussakis MN. Cell-autonomous epithelial activation of AIM2 (absent in melanoma-2) inflammasome by cytoplasmic DNA accumulations in primary Sjogren's syndrome. J Autoimmun. (2020) 108:102381. 10.1016/j.jaut.2019.102381 31919014

[70] Song Y Na HS Park E Park MH Lee HA Chung J. Streptococcus mutans activates the AIM2, NLRP3 and NLRC4 inflammasomes in human THP-1 macrophages. Int J Oral Sci. (2018) 10:23. 10.1038/s41368-018-0024-z 30078841 PMC6080406

